# Variation of stroke-associated pneumonia in stroke units across England and Wales: A registry-based cohort study

**DOI:** 10.1177/17474930211006297

**Published:** 2021-04-09

**Authors:** MA Lobo Chaves, Matthew Gittins, Benjamin Bray, Andy Vail, Craig J Smith

**Affiliations:** 1 Division of Cardiovascular Sciences, School of Medical Sciences, University of Manchester, Manchester, UK; 2 Centre for Biostatistics, University of Manchester, Manchester, UK; 3 School of Population Health and Environmental Sciences, King’s College London, London, UK; 4 Manchester Centre for Clinical Neurosciences, Salford Royal NHS Foundation Trust, Salford, UK

**Keywords:** Stroke, pneumonia, complications

## Abstract

**Background:**

Pneumonia is common in stroke patients and is associated with worse clinical outcomes. Prevalence of stroke-associated pneumonia varies between studies, and reasons for this variation remain unclear. We aimed to describe the variation of observed stroke-associated pneumonia in England and Wales and explore the influence of patient baseline characteristics on this variation.

**Methods:**

Patient data were obtained from the Sentinel Stroke National Audit Programme for all confirmed strokes between 1 April 2013 and 31 December 2018. Stroke-associated pneumonia was defined by new antibiotic initiation for pneumonia within the first seven days of admission. The probability of stroke-associated pneumonia occurrence within stroke units was estimated and compared using a multilevel mixed model with and without adjustment for patient-level characteristics at admission.

**Results:**

Of the 413,133 patients included, median National Institutes of Health Stroke Scale was 4 (IQR: 2–10) and 42.3% were aged over 80 years. Stroke-associated pneumonia was identified in 8.5% of patients. The median within stroke unit stroke-associated pneumonia prevalence was 8.5% (IQR: 6.1–11.5%) with a maximum of 21.4%. The mean and variance of the predicted stroke-associated pneumonia probability across stroke units decreased from 0.08 (0.68) to 0.05 (0.63) when adjusting for patient admission characteristics. This difference in the variance suggests that clinical characteristics account for 5% of the observed variation in stroke-associated pneumonia between units.

**Conclusions:**

Patient-level clinical characteristics contributed minimally to the observed variation of stroke-associated pneumonia between stroke units. Additional explanations for the observed variation in stroke-associated pneumonia need to be explored which could reduce variation in antibiotic use for stroke patients.

## Introduction

Stroke-associated pneumonia (SAP) is a complication defined as the spectrum of lower respiratory tract infection complicating the first seven days after stroke admission.^1,2^ It is associated with increased mortality, worse outcomes in survivors, and increased length of hospital stay.^3^ SAP occurs in around 14% of patients^4^ although there is marked variation in reported frequency between observational studies, registries, and within registries, where SAP frequency in observational studies ranged from 5.3 to 37.9% and in registries from 6.7 to 30%.^4,5^ The underlying reasons for this variation are uncertain, but could include potentially modifiable or non-modifiable factors. Baseline characteristics, such as increased stroke severity, increased age, and dysphagia,^5^ are consistently associated with SAP and might therefore contribute to variation in reported SAP frequency. Seasonality may also contribute to this variation.^6^ However, other factors contributing to variation in observed SAP between studies or units could be modifiable differences in care processes (e.g. monitoring of vitals or swallow screening protocols), approaches to diagnosis, and thresholds for initiating antibiotics (physician diagnosis vs. application of standardized algorithms).^7,8^

Better understanding the underlying variation in SAP is important if modifiable factors can be identified. This could lead to avoidance of over or under-treatment of SAP, improved antibiotic stewardship, reduced antibiotic resistance, and improved clinical outcomes. A key first step is determining how much variation is accounted for by non-modifiable clinical characteristics. The aim of this study is to therefore describe the variation of SAP between stroke units and determine how much this variation can be explained by patient baseline clinical characteristics. Our objectives were to first describe the observed variation of SAP across stroke units in England and Wales participating in a large national registry; and then to compare the estimated probabilities of developing SAP across stroke units adjusted and unadjusted for patient-level characteristics.

## Methodology

### Study design and data source

We undertook an observational cohort analysis, using anonymized, patient-level, and unit-level data from the Sentinel Stroke National Audit Programme (SSNAP) database. SSNAP is a national audit of stroke care created in 2013 in association with the Intercollegiate Stroke Working Party.^9^ Data transfer from SSNAP for the study was approved by the Healthcare Quality Improvement Partnership (HQIP). Data access requests should be directed to HQIP as the joint data controller and SSNAP as the data provider. All stroke units in England, Wales, and Northern Ireland are required to provide patient characteristics, processes of care, and specified outcome measures. We requested initial admission patient-level SSNAP recorded and derived variables. Patients were included if presenting with ischemic or hemorrhagic stroke to a stroke unit between 1 April 2013 and 31 December 2018 in England and Wales. Stroke units with <150 patients per year in the five-year period were excluded post-hoc in order to better reflect established specialist stroke units. Observed SAP was defined as those patients recorded as having new antibiotic initiation for pneumonia within the first seven days of stroke admission. Dysphagia was defined in patients with a baseline swallow screen followed either by a speech and language therapist (SLT) assessment or no SLT assessment due to being “too unwell” or for organizational reasons.^10^

### Statistical analysis

Descriptive statistics were used for baseline clinical characteristics, including the distribution of observed SAP cases per year across stroke units. Stroke patients are clustered within stroke units. To account for this lack of independence between stroke patients whilst accounting for confounding, a multilevel mixed effects logistic regression model was fitted. The random effects intercept present in the model accounts for the within and between variation present within the stroke unit clusters. Two models were fitted, one without (unadjusted) and one with (adjusted) fixed effect covariates. Through modeling only the stroke units as a random effects intercept, we estimated the predicted probability of SAP across stroke units without adjusting for clinical factors. To investigate the influence of stroke patient characteristics, the fixed effects covariates were identified a priori to include vascular and SAP risk factors at baseline entry. Vascular risk factors included the presence of hypertension, diabetes, and congestive heart failure. SAP risk factors included atrial fibrillation, previous stroke or transient ischemic attack (TIA), level of consciousness (LOC = 0–3, 3 is completely unconscious), total baseline National Institutes of Health Stroke Scale (NIHSS) score, pre-stroke modified Rankin Scale (mRS), sex, age on admission, and dysphagia.

Odds ratios (ORs) with 95% confidence intervals (CIs), intra-class correlation coefficient, and cluster variance were reported and compared for the two models where appropriate. Predicted probabilities from the unadjusted and adjusted model were calculated and ranked (smallest to largest) and plotted for the stroke units. Patients discharged from hospital care before seven days may subsequently develop SAP and thus be misclassified. We therefore performed an additional sensitivity analysis where patients’ length of stay was ≥7 days post first admission. Statistical analysis was performed using Stata IC version 14.

## Results

A total of 456,590 stroke patients across 322 units were identified, of which 39,397 (8.73%) had observed SAP after 5200 patients were excluded due to missing SAP identification data. A further 153 stroke units (38,257 patients) with ≤150 patients per year were excluded, leaving 413,133 patients of which 34,987 (8.47%) had observed SAP. A full description of baseline characteristics is presented in Table 1. Of the female patients, 17,980 (8.76%) developed SAP, while 17,007 (8.00%) of the male patients developed SAP, of those patients over 80 years of age, 21,359 (13.9%) developed SAP, 31,889 (8.53%) of patients of white ethnicity developed SAP, and 29,460 (8.04%) of patients admitted due to ischemic stroke developed SAP. The median admission NIHSS of patients who developed SAP was 14 (interquartile range (IQR): 6–20).

**Table 1.** Baseline characteristics of SAP patients

	non-SAP	SAP	Missing
Total number	378,146 (91.5%)	34,987 (8.47%)	4777 (1.14%)
Female	184,734 (90.0%)	17,980 (8.76%)	2524 (1.22%)
Male	193,412 (90.9%)	17,007 (8.00%)	2253 (1.05%)
Age on arrival (years)			
<60	58,604 (95.9%)	1846 (3.02%)	687 (1.12%)
60–69	63,839 (93.9%)	3395 (4.99%)	741 (1.09%)
70–79	102,229 (91.4%)	8397 (7.51%)	1182 (1.05%)
80–89	114,011 (87.5%)	14,646 (11.2%)	1571 (1.20%)
>90	39,463 (84.3%)	6713 (14.3%)	596 (1.27%)
Ethnicity			
White	337,526 (90.4%)	31,889 (8.53%)	4118 (1.10%)
Asian	10,919 (92.1%)	756 (6.38%)	177 (1.49%)
Black	4617 (93.7%)	252 (5.11%)	60 (1.21%)
Mixed	1272 (90.5%)	110 (7.82%)	24 (1.71%)
Other	23,812 (90.9%)	1980 (7.56%)	398 (1.52%)
CHF	19,022 (85.2%)	2986 (13.4%)	306 (1.37%)
Hypertension	202,701 (90.3%)	19,232 (8.57%)	2432 (1.08%)
Diabetes	78,158 (90.4%)	7390 (8.54%)	933 (1.07%)
Atrial fibrillation	71,285 (86.0%)	10,501 (12.7%)	1056 (1.27%)
Previous stroke or TIA	99,885(89.9%)	10,029 (9.03%)	1196 (1.08%)
Stroke subtype			
Infarction	333,119 (90.9%)	29,460 (8.04%)	3863 (1.05%)
Primary ICH	42,272 (87.3%)	5321(11.0%)	814 (1.68%)
mRS before stroke			
0	212,719 (93.1%)	13,156 (5.76%)	2524 (1.11%)
1	57,772 (90.4%)	5439 (8.51%)	680 (1.06%)
2	38,017 (87.9%)	4765 (11.0%)	485 (1.12%)
3	41,547 (85.4%)	6531 (13.4%)	581 (1.20%)
4	21,678 (83.6%)	3898 (15.0%)	360 (1.39%)
5	6413 (82.7%)	1198 (15.4%)	147 (1.89%)
NIHSS on arrival (median, IQR)	4 (2–9) *n* = 330,710	14 (6–20) *n* = 27,800	–
Level of consciousness			
0	326,642 (93.0%)	21,050 (6.00%)	3370 (0.96%)
1	29,579 (77.0%)	8223 (21.4%)	609 (1.59%)
2	12,384 (74.0%)	4025 (24.0%)	328 (2.00%)
3	9541 (81.5%)	1689 (14.4%)	470 (4.02%)
Dysphagic	14,394 (32.4%)	27,405 (61.7%)	2614 (5.89%)

CHF: congestive heart failure; TIA: transient ischemic attack; ICH: intracerebral hemorrhage; mRS: modified Rankin Scale; NIHSS: National Institutes of Health Stroke Scale; IQR: interquartile range; SAP: stroke-associated pneumonia.

**Figure 1.**
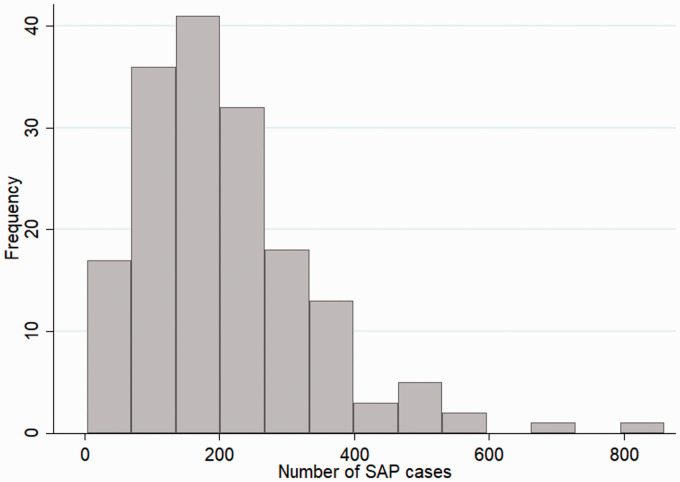
Histogram showing the distribution of the average number of observed SAP episodes per year for each stroke unit. SAP: stroke-associated pneumonia.

The total number of observed SAP episodes across 169 stroke units ranged from 4 to 860 for the five-year period. A histogram of the number of observed SAP episodes for each stroke unit is plotted in Figure 1. The median number of observed stroke unit SAP episodes per year was 29.8 (IQR: 20.8–44.5). The observed SAP prevalence per year ranged from 0.82 to 21.4%, with the median prevalence 8.50% (IQR: 6.06–11.5%).

**Figure 2.**
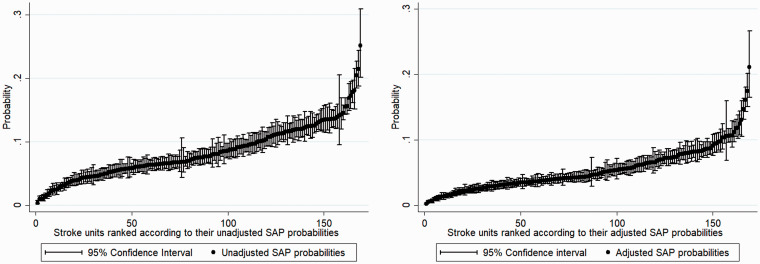
Stroke units ranked according to their (a) unadjusted SAP probabilities and (b) adjusted SAP probabilities with their corresponding 95% confidence intervals. SAP: stroke-associated pneumonia.

**Figure 3.**
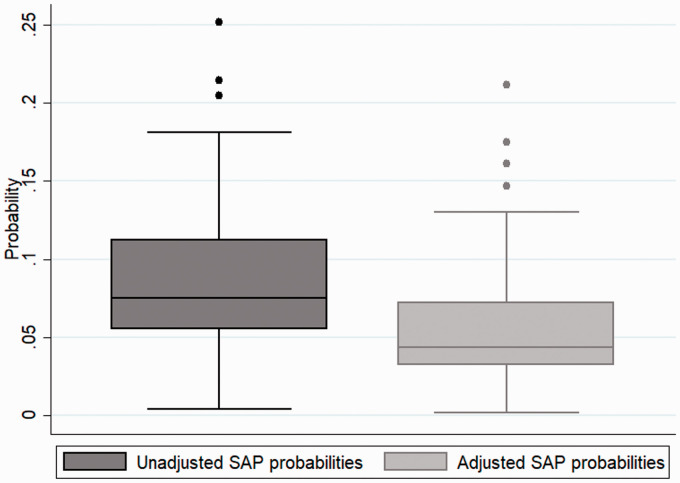
Box plots comparing unadjusted SAP probabilities vs adjusted SAP probabilities. SAP: stroke-associated pneumonia.

Both regression models were applied to the same complete cases population group of 341,740 patients across 169 units after accounting for missing data. Table 2 reports OR and 95% CIs from the adjusted mixed logistic regression model. Several factors were associated with SAP, including atrial fibrillation (OR: 1.2, 95% CI: 1.1–1.2), congestive heart failure (OR: 1.3, 95% CI: 1.2–1.4), dysphagia (OR: 3.8, 95% CI: 3.7–3.9), and NIHSS score on arrival (OR: 1.06, 95% CI: 1.06–1.07). The estimated predicted probability of SAP per stroke unit in the unadjusted model ranged from 0.004 to 0.25, compared to 0.001 to 0.21 in the adjusted model. The (unadjusted) median SAP probability was 0.07 (IQR: 0.05–0.11), whereas the adjusted was 0.04 (IQR: 0.03–0.07). The mean and variance of the predicted SAP probability across stroke units decreased from 0.08 (0.68) to 0.05 (0.63) when adjusting for patient admission characteristics. Unadjusted variance was 0.68 (95% CI: 0.53–0.89) and adjusted variance was 0.63 (95% CI: 0.549–0.81). This change in variance indicates that clinical characteristics account for 5% of observed variation. Figure 2(a) and (b) plot the predicted probabilities (and 95% CIs) across modeled stroke units for both models. Figure 3 presents the spread of the data for both the unadjusted and adjusted probabilities. Intraclass correlation value for the unadjusted model was 0.17 (95% CI: 0.14–0.21), while the intraclass correlation value for the adjusted model was 0.16 (95% CI: 0.13–0.20).

**Table 2.** Multivariable multilevel logistic regression odds ratios for the predictor variables for SAP

Predictors	Odds ratio	95% confidence interval	Unadjusted model	95% confidence interval
Age on arrival				
<60	1.00 (reference)	–	–	–
60–69	1.5	(1.4–1.6)	–	–
70–79	2.0	(1.9–2.1)	–	–
80–89	2.5	(2.3–2.6)	–	–
>90	2.5	(2.3–2.6)	–	–
Modified Rankin Scale score on arrival				
0	1.00 (reference)	–	–	–
1	1.3	(1.2–1.3)	–	–
2	1.4	(1.3–1.5)	–	–
3	1.4	(1.4–1.5)	–	–
4	1.3	(1.2–1.3)	–	–
5	1.1	(0.96–1.1)	–	–
Female sex	0.72	(0.71–0.75)	–	–
Atrial fibrillation	1.2	(1.1–1.2)	–	–
Previous stroke or TIA	0.92	(0.89–0.94)	–	–
Congestive heart failure	1.3	(1.2–1.4)	–	–
Hypertension	0.99	(0.96–1.0)	–	–
Diabetes	1.1	(1.0–1.1)	–	–
Level of consciousness				
0	1.00 (reference)	–	–	–
1	1.4	(1.4–1.5)	–	–
2	1.1	(1.0–1.1)	–	–
3	0.35	(0.32–0.38)	–	–
Dysphagia	3.9	(3.8–4.0)	–	–
NIHSS score	1.06	(1.05–1.07)	–	–
Variance	0.63	(0.49–0.81)	0.68	(0.52–0.89)
Random intercept coefficient	–4.4	(–4.5 to −4.3)	–2.2	(–2.3 to −2.2)
Intra-class correlation coefficient	0.16	(0.13–0.20)	0.17	(0.14–0.21)

TIA: transient ischemic attack; NIHSS: National Institute of Health Stroke Scale.

The number of patients who left hospital prior to seven days was 186,633 (45.2 %), leaving the sensitivity analysis with 33,076 (14.3%) patients with SAP and 195,074 (84.4%) non-SAP patients. Results from the sensitivity analysis (Table I in the online only Data Supplement) were comparable to our main findings, indicating misclassification of SAP patients was minimal.

## Discussion

Our findings show that there is substantial variation of observed SAP episodes, and therefore antibiotic use for pneumonia, across stroke units in England and Wales. This could be of major importance if it reflects under- or over-treatment with antibiotics. We also found that predicted probabilities of SAP were minimally modified after adjustment for patient-level clinical characteristics. This implies that differences in patient characteristics that are associated with risk of SAP contributed only marginally to the between unit variation in SAP. This is important because it suggests that additional, potentially modifiable factors account for most of the variation.

Several other factors could be important contributors to the observed variation in SAP. First, there is known variation in how clinicians diagnose SAP, with approaches ranging from non-standardized diagnosis (physician diagnosis) to application of various algorithm-based criteria such as the Centers for Diseases Control and Prevention criteria or the American Thoracic Society and Infection Diseases Society of America criteria.^4^ This is of relevance as clinicians tend to overdiagnose SAP when compared to application of an established algorithm.^8^ There is also evidence that the thresholds for diagnosing SAP and initiating antibiotics is linked to clinician behaviors, with weighting toward particular variables such as stroke severity and C-reactive protein concentration.^7^ Perceived futility in patients with severe stroke and multiple comorbidities could lead to clinicians withholding antibiotics. Secondly, seasonality could also influence this variation. The microbiological etiology of SAP incorporates organisms associated both with community-acquired pneumonia (CAP) and hospital-acquired pneumonia.^11^ CAP is frequently viral, and viral contributions to SAP linked to seasonal occurrence of CAP or viral lower respiratory tract infections have not been investigated. A third potential explanation could be differences in stroke unit care and processes between stroke units. Several components of stroke care, and how quickly, consistently, or effectively they are delivered may influence development of SAP. For example, delays in dysphagia assessment have been associated with increased risk of SAP.^12^ High-volume thrombolysis and thrombectomy units with good outcomes are likely to be associated with lower incidence of SAP.^13,14^ Further evaluation is needed on the impact of multi-disciplinary team interventions such as positioning, nasogastric tube feeding, or early mobilization on SAP development,^15^ as well as the impact of organizational level factors such as staffing levels.

Several patient-level characteristics included in this study were associated with increased odds of SAP, among them were NIHSS score and LOC, increased age, higher preceding modified mRS score, congestive heart failure, diabetes, atrial fibrillation, and dysphagia. These characteristics have already been described in previous studies and generally found to be associated with increased risk of SAP.^16–18^ Our findings are consistent with previous research, which highlight the importance of these clinical predictors. However, an unexpected finding was that previous stroke or TIA was not associated with SAP. This contrasts with previous evidence,^19^ and the reasons for this in our study are not clear and should be interpreted with caution. One possible issue is the heterogeneity of the variable, as previous TIA or stroke may affect the risk of developing SAP differently but was not differentiated. Important factors such as the subtype, occurrence of >1 previous stroke, residual neurological deficit, and when the previous event occurred were not recorded. Another possible explanation for our findings is the Table 2 fallacy.^20^ This is where the effects estimates that we are reporting are those of our SAP outcome variable and do not represent the effect of previous stroke or TIA that has also undergone stroke severity adjustment. This is the Table 2 fallacy, where there is interpretation of direct effects as total effects. Such effect estimates represent the direct effect between the covariate and outcome and is not representative of the total effect of the confounder, which may include the “indirect” effect between confounder and outcome via the main predictor. In our specific case with previous stroke, the “total” effect of this covariate may be comprised of the “direct” effect with SAP outcome and the “indirect” effect via stroke severity at baseline, as previous stroke is likely to be correlated with both.

The clinical impact and implications of our findings require consideration. SAP is consistently associated with adverse clinical outcomes, including increased mortality, healthcare costs, and worse functional outcome in survivors.^1,3^ Antibiotic usage, and in particular concerns around antibiotic over-use and antimicrobial resistance, are major global public health concerns.^18^ However, under-treatment of SAP may also impact clinical outcomes. How our observed variation in SAP episodes relates to clinical outcomes is unclear and requires further study. Strategies to reduce over-diagnosis or treatment of SAP, whilst not compromising clinical outcomes, could have significant impact on antibiotic stewardship, reducing antibiotic and healthcare costs, and antimicrobial resistance.^21^ Identification of other stroke unit practices or interventions associated with variation in SAP could stimulate quality improvement initiatives or trials.

Our study contains several strengths, including a large sample size over several years and a large proportion of the target stroke population, with high case ascertainment, improving the generalizability of our findings. SSNAP also records a number of patient characteristics that are reproducible clinical predictors of SAP.^16,17^ However, our study also has several limitations. First, there is no record of diagnostic approach or decision-making processes used by clinicians to initiate antibiotic treatment and therefore observed SAP episodes. Second, methods for capturing SAP episodes for SSNAP entry may differ between units which could lead to under-reporting relating to consistency of coding. As SAP episodes in SSNAP are based on new antibiotic initiation, withholding antibiotics due to perceived futility in patients with severe stroke and a clinical diagnosis of SAP would also lead to underestimates of SAP frequency. There are also limitations within the completeness of the dataset in several measured variables with the most notable being the NIHSS score. Another is the reliability of some of the included variables, particularly derived dysphagia status. As dysphagia is not recorded directly in SSNAP, derivation of dysphagia could have misclassified some patients. The patient-level clinical characteristics in our models were limited by the variables recorded in SSNAP, or derived from them. Several variables that have been associated with SAP are not recorded in SSNAP, most notably chronic obstructive pulmonary disease and smoking status. These two characteristics have previously been associated with increased risk of developing SAP,^17^ and could have contributed to the variation in SAP between stroke units. Other variables such as oncological status, immunological status, and vascular risk factors which have been associated with pneumonia are not recorded in SSNAP,^22,23^ and could limit our conclusions.

## Conclusions

Large variation of SAP, and therefore antibiotic use, has been observed between stroke units during a five-year period in England and Wales. This variation can be accounted for, to a limited extent, by patient-level clinical characteristics alone. However, further research is needed to determine additional factors contributing to this variation, such as diagnostic approach, clinician behaviors, organizational aspects of stroke care, patient care processes, seasonal factors, and potentially modifiable factors that could have an impact on SAP variation.
